# The *Dictyostelium* Centrosome

**DOI:** 10.3390/cells10102657

**Published:** 2021-10-05

**Authors:** Ralph Gräf, Marianne Grafe, Irene Meyer, Kristina Mitic, Valentin Pitzen

**Affiliations:** Department of Cell Biology, University of Potsdam, Karl-Liebknecht-Str. 24–25, 14476 Potsdam-Golm, Germany; mgrafe@uni-potsdam.de (M.G.); irene.meyer@uni-potsdam.de (I.M.); mitic@uni-potsdam.de (K.M.); valentin.pitzen@uni-potsdam.de (V.P.)

**Keywords:** microtubule-organizing center, microtubule-organization, centrosome, *Dictyostelium*, mitosis

## Abstract

The centrosome of *Dictyostelium* amoebae contains no centrioles and consists of a cylindrical layered core structure surrounded by a corona harboring microtubule-nucleating γ-tubulin complexes. It is the major centrosomal model beyond animals and yeasts. Proteomics, protein interaction studies by BioID and superresolution microscopy methods led to considerable progress in our understanding of the composition, structure and function of this centrosome type. We discuss all currently known components of the *Dictyostelium* centrosome in comparison to other centrosomes of animals and yeasts.

## 1. Introduction

### 1.1. Centrosome Types and Centrosome Duplication

**Figure 1:**
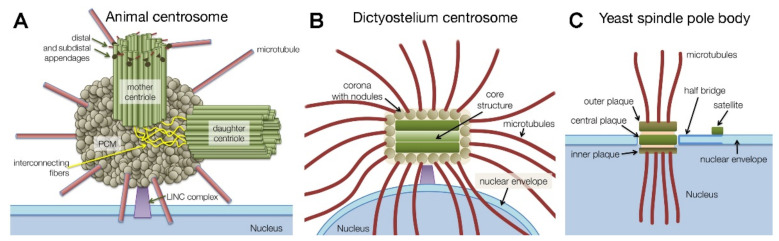
Schematic comparison of centrosomal structures in animals (**A**), *Dictyostelium* (**B**), and budding yeast (**C**). Functionally or topologically related structures are drawn in corresponding colors. Taken from [4].

Centrosomes are proteinacious organelles best known for their function as major microtubule organizing centers (MTOCs). They have been extensively studied since the late 19th century, when they were first characterized independently by three pioneers, Walther Flemming, Theodor Boveri and Edouard van Beneden [1,2,3,4]. While studying cell division in various fertilized eggs and tissues they recognized a role of centrosomes in mitotic spindle formation and chromosome movements. Although it quickly became clear that centrosomes duplicate once per cell cycle and that they nucleate and organize microtubules, it took until the late eighties of the last century to gain more insight into the manner in which centrosomes manage to do so, when γ-tubulin was identified as a third tubulin isoform required for microtubule nucleation [5]. At that time, it also became apparent that centrosomes consist solely of proteins, and—besides kinetochores—represent the largest and most complicated protein complex in a eukaryotic cell, within the order of 100 different protein components [6]. Comparative evolutional biology revealed that precursors of centrosomes were already a feature of the last eukaryotic common ancestor (LECA) [7,8,9]. During evolution different centrosome types emerged (Figure 1), and in a few branches of the eukaryotic tree of life, centrosomes were even lost, most prominently in higher plants. The most common type of centrosome is characterized by the presence of centrioles, which consist of a nine-fold symmetric cylindrical assembly of short microtubules [10]. In G1, there is one older, mother centriole, and one younger, daughter centriole. Mainly the mother centriole is embedded in a highly structured pericentriolar matrix, which contains the majority of the microtubule-nucleating γ-tubulin ring complexes [11]. Centrosomal organelles replicate once and only once per cell cycle, ensuring that after mitosis, mononucleated cells always contain only one single centrosomal entity. In mammalian cells, in late mitosis Polo-like kinase 1 and separase play a key role in licensing each of the two centrioles to a further round of duplication in the following cell cycle [12]. 

Centriole duplication is then initiated in synchrony with DNA replication through the action of cyclin-dependent kinase 2 (CDK2) [13]. The assembly of procentrioles at the side walls of mother and daughter centrioles requires active Polo-like kinase 4 (Plk4) [14]. Plk4 is recruited to a Cep192 sleeve around the parent centrioles and phosphorylates STIL, which recruits CPAP to STIL. Furthermore, STIL phosphorylation recruits SAS6, which forms the 9-fold symmetric precursor of the so-called cartwheel at the side walls of mother and daughter centrioles [14,15]. Cartwheel formation includes recruitment of further proteins including the spoke head protein Cep135 and finally the nucleation and binding of the centriolar microtubules [16]. Together, the cartwheel and microtubules make up the procentrioles. Centriolar microtubules grow until the procentrioles have reached almost the same length as the parent centrioles. In late G2, Plk1, Cep192 and Aurora A kinase promote the growth of the pericentrosomal matrix and thus, increased microtubule nucleation [17]. At this time, mother and daughter centrioles, each equipped with an almost mature procentriole, are still interconnected by fibers involving rootletin, Cep68, centlein, LRRC45, CDK5RAP2, GAS2L1 and C-Nap1/Cep215 [18,19,20,21,22,23]. Phosphorylation of these proteins by the NIMA-related kinase Nek2 causes the disassembly of the interconnecting fibers and allows the two centrosomal entities to move apart and form the two opposing spindle poles [24]. During late mitosis the orthogonal orientation of the former procentrioles to their parent centrioles is released through the activity of Plk1 and the cystein protease separase [14]. This event is called disengagement and primes each centriole for a new round of centriole duplication. To serve as a new parent centriole, the former procentriole undergoes centriole maturation, a process again regulated by Plk1 and leading to the recruitment of Cep192 and CDK5RAP2 [25].

Mother centrioles also serve as precursors of basal bodies of primary cilia. Thus, centriole-containing centrosomes are found in all organisms capable of forming cilia, at least in specific cell types or developmental stages. On the other hand, acentriolar centrosomes are typically found in organisms lacking cilia, including amoebozoans and many fungi [9]. Acentriolar centrosomes have been intensely studied in yeast, where they are called spindle pole bodies (SPBs), and in the amoebozoan model organism *Dictyostelium discoideum*, where the centrosome is also known as nucleus-associated body (NAB) [8,26]. Since they are evolutionary related organelles serving the same function, in this review we will call all these organelles centrosomes. While fungi and animals are in the same eukaryotic supergroup Opisthokonta, the *Dictyostelium* centrosome is the only well-established model for an acentriolar centrosome outside the Opisthokonta. Acentriolar centrosomes are usually characterized by a stack of electron-dense, plaque-like protein assemblies that during interphase are either embedded in a fenestra of the nuclear envelope (budding yeast) or attached to the cytosolic face of the nucleus (fission yeast, *Dictyostelium*) (Figure 1). The *Dictyostelium* centrosome consists of a cylindrical core structure with three major layers surrounded by a corona, in which γ-tubulin containing nodules are embedded [27,28,29]. The whole structure resembles an ellipsoid with a diameter of ~500 nm along its long axis. The layered structures in yeasts and *Dictyostelium* are most likely analogous but not homologous, due to differences in biogenesis during the process of centrosome duplication. While in yeast new spindle pole bodies are formed de novo starting with the assembly of a so-called satellite at the distal end of a bridge-like extension of the old spindle pole body [30], duplication of the *Dictyostelium* centrosome occurs in a semiconservative manner, in which each new centrosome shares equal parts of the former old centrosome [30,31].

**Figure 2:**
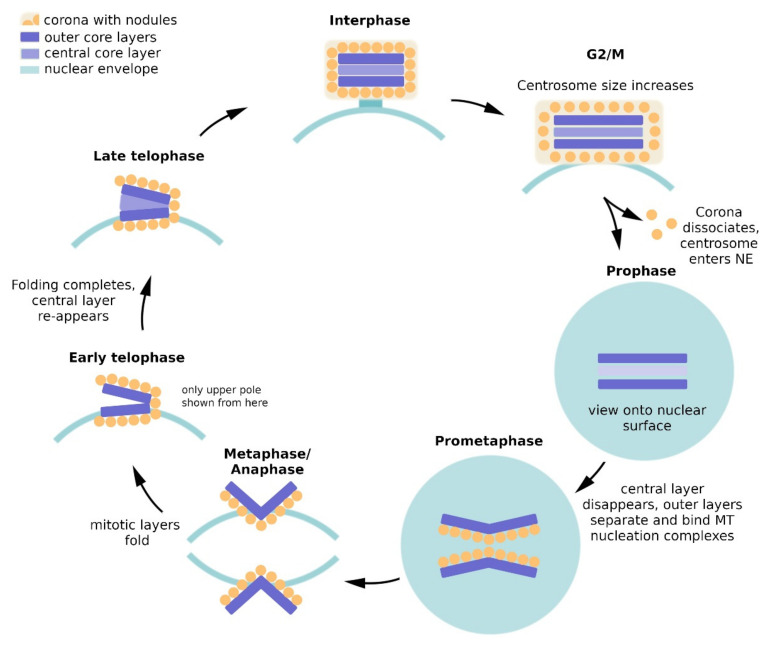
The *Dictyostelium* centrosome cycle. Nuclei and centrosomes are shown in schematic cross sections, except for the prophase and prometaphase images where a surface view is shown. See text for a detailed description. Redrawn and adapted from [33].

*Dictyostelium* centrosome duplication starts at the G2/M transition (Figure 2) [31]. First the size of the whole centrosome increases in all dimensions and the corona dissociates, along with the microtubule-nucleation complexes. This is accompanied by the disassembly of all pre-existing microtubules. Next, the remaining core structure enters the nuclear envelope, and the central core layer disappears. In prometaphase the outer core layers start to separate, each one residing in its own fenestra of the nuclear envelope. According to our current knowledge (K. Mitic, P. Batsios and R. Gräf, unpublished results) the nuclear envelope becomes leaky at the fenestrae harboring the mitotic centrosomes, allowing the exchange of spindle assembly factors and tubulin dimers. This type of mitosis without nuclear envelope breakdown, instead featuring a leaky nuclear envelope, is called a semi-closed or semi-open mitosis [32].

The former outer core layers act as mitotic centrosomes, and upon their separation they nucleate spindle microtubules forming a central spindle. In metaphase, astral microtubules appear. Starting with anaphase, the plaque-shaped mitotic centrosomes undergo a folding process, in which the inner, microtubule-nucleating surface becomes increasingly exposed to the cytoplasm. In telophase, the folding process of each mitotic centrosome completes with a scission at the kink of the fold, and the re-appearance of the central core layer. This process implicates an inside-to-outside reversal of the outer core layers in each cell cycle [31] and suggests that the two outer core layers have the same protein composition. The new centrosomes then exit their fenestrae in the nuclear envelope but remain attached to the cytosolic surface of the nucleus via a connector including the nuclear envelope protein Sun1. At this time the microtubule nucleating surface of the new core structure differentiates into the new corona.

### 1.2. Centrosome Functions

The most obvious function of centrosomes is to serve as the major microtubule organizing center (MTOC) during the entire cell cycle. Consequently, after they have duplicated exactly once in the previous cell cycle, mitotic centrosomes form the poles of the mitotic spindle. Since the times of Boveri, Flemming and van Beneden the centrosome’s role as the organizer of the mitotic spindle had been considered the key function. However, this view was challenged after researchers realized that there are cells capable of undergoing mitosis without centrosomes, as for instance in early rodent embryos or in several cell lines, e. g., from *Drosophila*. Furthermore, laser ablation experiments and studies employing *Xenopus* egg extracts clearly showed that for bipolar spindle formation, centrosomes are dispensable [34,35]. This is due to the existence of a pathway for microtubule nucleation in the absence of centrosomes. Here, spindle microtubules are nucleated in the vicinity of chromatin, by a pathway employing Ran-GTP, TPX2 and Aurora A, together with spindle assembly factors (SAFs) [36,37]. Moreover, microtubules are augmented by further microtubule nucleation via the augmin/HAUS complex, which binds γ-tubulin complexes at pre-existing microtubules [38]. All mitotic microtubules are then sorted and oriented through the activity of kinesins and dynein/dynactin to form a bipolar spindle. Assessed by the widespread conservation of its key components, this acentrosomal spindle assembly mechanism should have been present already in the LECA. However, if and when centrosomes are present, they will also participate in bipolar spindle formation, and if present in the wrong number they will interfere with spindle formation, as for example in most tumor cells [12].

The dispensability of centrosomes for bipolar spindle formation in many cell types raised the question what they are good for in these cells. Of course, their function as MTOCs in interphase cells and resulting role in organelle positioning along microtubules is obvious. Yet, diligent analysis of the fates of cells after laser ablation of mitotic centrosomes and the properties of cells with acentrosomal spindles revealed further functions. Centrosomes turned out as a strict requirement for the formation of astral microtubules during mitosis. These microtubules connect the spindle poles to the peripheral cell cortex and play a crucial role in cytokinesis. A subset of astral microtubules is essential for RhoA activation in order to induce recruitment of the contractile actin/myosin ring and, thus, cleavage furrow formation [39]. Furthermore, centrioles are involved in the deposition of at least two centrosomal proteins at the midbody, Cep55 and centriolin. The latter is a mammalian homologue of budding yeast Nud1p and fission yeast Cdc11, which stand at the beginning of the mitotic exit network (MEN) and septation initiation network (SIN), respectively [40]. Assembly of these proteins at the midbody drives the abscission between the two daughter cells through recruitment of the ESCRT complex and Golgi vesicles [41,42,43,44]. After passage through cytokinesis, intact centrosomes are required for passage through G1 [45] the mother centriole acts as the basal body for the formation of the primary cilium [46]

## 2. *Dictyostelium* Centrosome Composition and Topology

In recent years, for the yeast SPB and the mammalian centrosome a fairly clear picture has emerged of the centrosomal composition and the subcentrosomal topology of individual protein components. This progress was specifically promoted by the availability of superresolution fluorescence microscopy methods, especially single particle localization microscopy (SPLM), stimulated emission depletion microscopy (STED) and expansion microscopy (ExM) (for a review on superresolution methods see [47]), and of advanced techniques to study protein-protein interactions. Methods such as proximitiy-dependent biotin identification (BioID), focused yeast two-hybrid screening (Y2H) and tandem-affinity purification (TAP) [48,49,50] led to deeper insights into the centrosomal interactome in animal centrosomes and budding yeast spindle pole bodies. 

**Figure 3:**
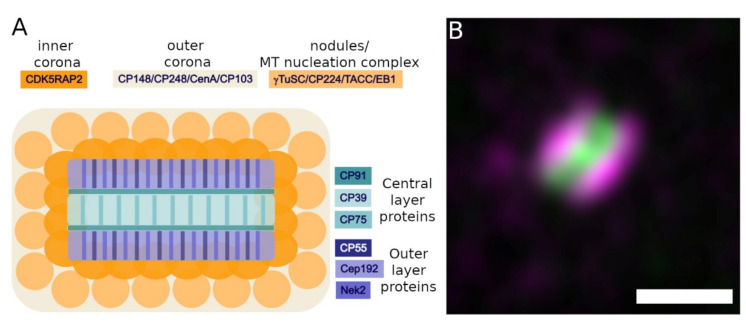
(**A**) Schematic of subcentrosomal distribution of the major centrosome components. The color code given with the protein names refers to the individual centrosomal substructures; (**B**) Expansion microscopy of nanobody-labeled Cep192 (magenta) and CP39 (green). Bar = 0.25 µm.

Meanwhile, also in the amoebozoan *Dictyostelium* model we have made considerable progress in the identification of centrosomal proteins, their subcentrosomal topology and interactions. After establishment of a centrosome isolation procedure [51], proteome analysis mainly of isolated centrosomes [52] and database mining led to the identification of currently 42 centrosomal and centrosome-associated proteins. The majority of them were assigned to centrosomal substructures by light and electron microscopy and, in many cases, their mutual interactions were further elucidated by TAP, BioID and co-precipitation analyses. A synopsis is given in Table 1 and Figure 3 and discussed in more detail in the following paragraphs. The protein names were usually taken from their best investigated orthologues at the time of their discovery. Proteins without obvious orthologues at the time of their discovery received a name with the abbreviation CP (centrosomal protein) and a number referring to their calculated molecular mass.

The proteins listed in Table 1 have been localized to the centrosome and/or are otherwise involved in centrosome function. This does not mean they are all required to maintain the structural integrity of the centrosome. Some proteins may only be temporary guests at the centrosome, either as regulators or using the centrosome as a hub for their own distribution within the cell via motor protein-dependent transport along microtubules.

Dynein, dynactin and dynein-regulators such as LIS1 are concentrated at centrosomes owing to the microtubule minus end-directed motor activity of dynein. This also causes a clustering of dynein cargos at the centrosome. The most obvious example in this respect is the Golgi apparatus, which is arranged around the centrosome due to the association of Golgi cisternae with dynein/dynactin [103,176]. Since its association with the centrosome is even detectable in isolated centrosomes devoid of microtubules, the dynein/dynactin/LIS1 complex may have additional binding partners among the centrosomal corona proteins. Microtubule-independent presence at the centrosome is a useful criterion to define a *bona fide* centrosomal protein and thus it was applied in *Dictyostelium* and other systems [177]. Therefore, the dynein complex proteins are also listed in Table 1, but no Golgi cargoes which are obviously lost upon the chemical and mechanical treatments during centrosome isolation [51]. 

In previous publications by us and others the *Dictyostelium* centrosome was subdivided into the corona, the outer core layers, and the central core layer, based both on light microscopy and behavior during mitosis. When stained with specific antibodies or expressed as GFP fusion proteins, in optical sections after deconvolution corona proteins show a ring-like appearance, with a ring diameter around 0.5 µm. Core proteins show spot-like stainings with no intensity gap in the center. Using conventional light microscopy, distinguishing between central and outer core layer proteins is beyond the resolution limit. Thus, proteins disappearing during mitosis were considered central core layer components, as the disappearance of the central layer was proven by electron microscopy [31], and permanent centrosomal residents were considered outer core layer proteins. We are aware that this categorization may be an over-simplification. Electron microscopy has shown that the corona contains nodules as a further substructure, and recent superresolution light microscopy data indicate that it can be subdivided in at least two distinct sheaths, one adjacent to the layered core and mainly consisting of CDK5RAP2, and another, distal sheath containing the majority of other corona proteins [54]. Also, sublayers exist within the three major layers of the core structure [27,28]. Moreover, it cannot be excluded that there are outer core layer proteins that are absent from mitotic spindle poles. However, despite its weaknesses, for practical reasons we will maintain the simplified categorization and provide more precise information where necessary.

### 2.1. Composition of the Corona

#### 2.1.1. γ-Tubulin and Its Interactors

γ-Tubulin is a prominent part of the corona. It was localized to the electron dense nodules by immuno-EM [29]. Although not proven by EM, it is conceivable that the other members of the γ-tubulin complex (γ-TuC), Spc97 and Spc98, are also present in the nodules [65]. Further members of the γ-tubulin ring complex (γ-TuRC) in animal cells, i.e., GCP4, GCP5, GCP6, GCP8/MZT2 and MZT1 [11,178], appear to be absent from the *Dictyostelium* genome. Thus, it is likely that like yeast, *Dictyostelium*, possesses only the small γ-tubulin complex (called γ-TuSC in animal cells), which forms ring-shaped arrangements only when associating with a γ-TuSC scaffolding protein [179]. In budding yeast this job is fullfilled by the pericentrin-like Spc110p on the nuclear side, and the CDK5RAP2-like Spc72p on the cytosolic side. Similarly, in fission yeast the respective orthologues Pcp1 and Mto1 are involved (Table 1 [179]). A further well known γ-TuRC binding protein in the pericentriolar matrix of animal cells, NEDD1/GCP-WD, is absent in yeasts.

*Dictyostelium* appears to employ the orthologues of the same proteins as γ-TuC scaffolding proteins as the two yeasts, i.e., CDK5RAP2 and CP148 [71,75]. CP148 should be considered the *Dictyostelium* orthologue of the Pericentrin (PCNT) family. These a-helical coiled coil proteins are present in all organisms possessing centrosomes, but only weakly conserved with regard to size and amino acid sequence similarity. CP148 is the best candidate for a pericentrin/kendrin/Spc110 orthologue in *Dictyostelium,* not only based on the a-helical coiled coil domains, some degree of sequence similarity, and the presence of a characteristic CaM-binding IQ-domain, but also with regard to its function and mutant phenotypes. Overexpression of CP148 results in a hypertrophy of the corona, while its depletion by RNAi causes a typical disintegration of the corona with dispersal of γ-tubulin containing microtubule-nucleation complexes [75]. However, during mitosis, CP148 is absent from spindle poles and dispensable for nucleation of spindle microtubules. This also indicates that the lining of MT nucleation complexes on top of the mitotic former outer layer, i.e., the mitotic centrosomes, is not simply the precursor of the new corona, because the latter *does* require CP148 for its integrity. Rather it is conceivable that this lining of mitotic microtubule-nucleation complexes undergoes a differentiation process to build the new corona, which involves the recruitment of CP148. This behavior of CP148 stands in contrast to CDK5RAP2 (also called Cep161 in *Dictyostelium* [180]) the second scaffolding protein for γ-TuCs, which is required for spindle formation [71]. CDK5RAP2 is absent from the centrosome only briefly in prophase upon disintegration of the corona but re-appears as soon as spindle microtubules are nucleated. As in case of CP148, depletion of CDK5RAP2 causes disintegration of the corona and the appearance of multiple, cytosolic microtubule nucleation complexes [71]. Superresolution microscopy indicated that it forms the interface between the corona and the layered core, since its localization closely matches that of the outer core layer component Cep192 [54]. 

#### 2.1.2. Centrosomal Microtubule-Associated Proteins

In animal cells CDK5RAP2/Cep215 serves as a platform for molecules important for the organization of mitotic spindle poles, through the presence of multiple binding domains for PCNT, γ-tubulin, Cep192, phosphorylated Aurora A, and motor proteins [181,182]. By analogy, *Dictyostelium* CDK5RAP2 could recruit not only CP148 and γ-TuCs but also the dynein complex (including dynein, dynactin and LIS1), CP224 (XMAP215 family), TACC (transforming acidic coiled coil protein), EB1 and CP248, which are all associated with the corona [64,78,80,86,103,109,180,183]. While the dynein complex is also associated with animal centrosomes, it has a specifically tight connection with the centrosome in *Dictyostelium*, which is independent of microtubules [103,109]. The same holds true for the microtubule plus-end associated proteins CP224, TACC and EB1, which mutually interact in tandem-affinity purification assays [184] and co-precipitate with components of the dynein complex [103,109]. The behavior of CP224, TACC and EB1 as centrosomal residents is not surprising, since the CP224 orthologue XMAP215 and TACC are not only promoters of microtubule growth but also involved in microtubule nucleation. This was first shown for Ran-GTP-driven acentrosomal spindle formation [185,186]. Recently, it was even shown that within γTuRCs XMAP215 interacts directly with γ-tubulin via its C-terminus, while its a/b-tubulin-binding TOG-domains supply the tubulin dimers for the nucleation of the filament [187]. The highly conserved EB1 protein is a centrosomal resident also of animal centrosomes [188], and in *Dictyostelium* it is required for proper spindle formation during prometaphase. EB1 knockout cells show a characteristically prolonged prometaphase [86]. In fission yeast, the EB1 orthologue Mal3 is associated with another microtubule-associated protein Moe1, which is also involved in mitotic spindle formation [92]. Moe1 is homologous to subunit 7 of the eukaryotic translation initiation factor 3 (also known as eIF-3z or eIF3d). Yet, so far, a role of eIF-3z in spindle formation has not been reported in any other organism. Interestingly, *Dictyostelium* Moe1 was localized to the centrosome using specific antibodies, and when expressed as a GFP-fusion protein it was also present at the corona of isolated centrosomes [91]. However, an interaction with EB1 or cooperation with EB1 in spindle formation up to now has not been verified in *Dictyostelium*. 

#### 2.1.3. Further Prominent Corona Components

Another interactor of CDK5RAP2 is CP248 (also called CP250), as shown by co-immunoprecipitation of both proteins [180]. CP248 was the first centrosomal protein to be identified in *Dictyostelium*. It was initially found using the NAB350 monoclonal antibody, which was also used to locate its antigen at the outer corona [189]. Only after our centrosomal proteome analysis had revealed CP248 [52] we found that this was the antigen for the NAB350 antibody. The NAB350 antigen was also assigned to the mitotic spindle [189], however later studies revealed that this was due to an unspecific secondary antibody. In fact, CP248 is absent from the spindle and mitotic centrosomes, as expected for a typical corona protein [64,93]. Independently, CP248 was found to be an interactor of the cyclase-activating protein CAP by yeast two-hybrid screening [93,190]. Although CAP deficiency elicited supernumerary centrosomes [93] the significance of the CAP-CP248 interaction remains unclear. However, CAP is absent from the centrosome and there is no functional relationship between adenylate cyclase activity and the centrosome. CAP is also known as an actin regulator and indeed, functional relationships between actin and centrosomes are known in animal cells. For example, γ-actin is important for early centrosome separation prior to mitosis [191]. Moreover, the centrosome acts as an actin filament organizer through the nucleation-promoter WASH in combination with the Arp2/3 complex and the PCM component PCM1 [192]. Despite the lack of a PCM1 orthologue, the latter function could be conserved in *Dictyostelium* as well, since a recruiter of WASH, HSBP1, has recently been detected at the *Dictyostelium* centrosome [143]. CP248 is one of the few centrosomal proteins a knockout of which appears to be viable [93]. The knockout strain shows only mild defects, with slight impairment of growth and development but no obvious defects in centrosome function. The only centrosome-related effect of CP248 deficiency was a reduced amount of Sun1 at the nuclear envelope. Sun1 is crucial for centrosome-nucleus attachment (see below), but surprisingly no respective defects have been described in CP248 knockout cells [93]. Yet one caveat remains. The knockout construct for homologous recombination was constructed in a way that it cannot be excluded that the resulting knockout cells still express an N-terminal part of the protein of 90 kDa [93].

There are several indications that CP248 could be an orthologue of C-Nap1 of animal cells [193]. C-Nap1, also known as Cep250) is a coiled coil protein at the proximal end of mother and daughter centrioles, where it is required for centriole cohesion. In late G2 it is phosphorylated by the NIMA-related kinase Nek2, causing its dissociation from centrioles along with the separation of the two centriole pairs later forming the spindle poles [94]. By analogy, CP248 could be required for in corona cohesion, in other words, dissociation of CP248 after phosphorylation by Nek2 could trigger dissociation of the corona at the G2/M transition. This idea is supported not only by structural similarities between CP248 and Cep250/C-Nap1 with regard to size and coiled coil structures, but also by immunological evidence, since C-Nap1-specific antibodies recognized CP248 purified from *Dictyostelium* [193]. However, whether CP248 is actually a substrate of Nek2 remains unknown. As with many coiled coil proteins, amino acid similarities are too weak to assess the degree of homology between the Cep250/C-Nap1 and CP248. The fact that knockout of CP248 does not grossly affect *Dictyostelium* centrosome structure or function, does not necessarily contradict this idea. In animal cells C-Nap1 is not the only protein involved in centriole cohesion, which needs to be phosphorylated by Nek2 to allow separation of the two centrosomal entities (see above [24]). If, in analogy, further components are required to be phosphorylated by Nek2 also in *Dictyostelium*, to allow the dissociation of the corona in prophase, the lack of only one component does not necessarily cause a readily detectable centrosomal phenotype. Likely candidates for further Nek2 substrates in this context are among the central core layer proteins (see below and [53]).

Despite its early identification, centrin still remains one of the most puzzling corona components [95]. Yeast centrin (Cdc31p) was the first centrosomal protein to be described on the molecular level [97]. Later, centrin orthologues were characterized as centrosomal components in all organisms containing this organelle. Yet, it has to be kept in mind that in many cell types, for instance human lymphoblasts, the major fraction of centrin is not centrosomal but located elsewhere in the cell, due to centrosome-independent functions such as nucleotide excision repair via the xeroderma pigmentosum group C complex (XPC), or the regulation of proteasome activity [194]. Centrins are small, calmodulin-like EF-hand proteins. Aside from yeast where Cdc31p is a member of the half-bridge and involved in satellite assembly during biogenesis of a new spindle pole body in interaction with Sfi1p [195], the centrosomal functions of its orthologues are less clear. Although centrins play a role in centriole duplication, they are not essential for this process (reviewed by [194]). In some organisms such as Xenopus, mouse and humans there are up to four different centrin isoforms, two of which are centriole-associated. *Dictyostelium* contains two isoforms, CenA (originally called DdCrp) and CenB, both of which are divergent compared to the four common centrin isoforms. Phylogenetic analysis revealed that they form their own clade [196]. CenA is localized at the centrosomal corona and is also present at mitotic centrosomes [95]. Although corona components are usually absent from mitotic spindle poles this is not without precedent. CDK5RAP2, as mentioned above, leaves the centrosome for a very brief period upon dissociation of the corona in prophase, then re-associates with mitotic spindle poles during spindle formation [71]. Interestingly, CenA was also found at the centromeres during interphase and mitosis. The functions of CenA are not known, neither at the centrosome nor at centromeres. The other centrin, CenB, turned out to be a nuclear protein. Interestingly, CenB knockout cells often contain supernumerary MTOCs, in addition to deformed nuclei, cytokinesis defects, and a disrupted centrosome-nucleus linkage. Altogether this suggested that CenB is somehow involved in the centrosome duplication cycle [196,197]. However, since CenB is absent from centrosomes throughout the entire cell cycle, this has to be an indirect role.

A still open question is the role of calcium in the regulation of centrins and centrosome function. In general, centrins are capable of binding calcium through their EF-hands. But there are only a few examples where a regulatory role of calcium has been demonstrated. For example, calcium binding to centrin regulates flagellar excision in green algae [198], and calcium binding to centrin1 regulates photoreceptor signaling in animals [199]. Calcium certainly plays a role in centrosome function, but more apparently through calmodulin and not via centrins. Calmodulin-dependent protein kinase II (CaMKII) regulates centrosome duplication along with CDK2 [200], Mps1 [201], polo-like kinases and Aurora kinases [202]. Furthermore, calmodulin is associated with centrosomes in several species. For example, it is a constituent of the central plaque of the yeast spindle pole body, and in mast cells it was found at mitotic spindle poles [203,204]. In *Dictyostelium*, calmodulin was found associated with the contractile vacuole during interphase and with the mitotic spindle during metaphase [205]. Calcium could also have regulatory roles via CP148, which contains a predicted EF-hand and calmodulin binding site (see above).

The last corona protein to discuss is CP103, a 103 kDa protein containing a domain characteristic of ZW10 proteins (Zeste white 10), a family of conserved, dynein-associated kinetochore proteins involved in regulation of the spindle assembly checkpoint. When expressed as a GFP-fusion protein CP103 localized to isolated, microtubule-free centrosomes, to the centrosomal corona and to spindle poles during metaphase but was absent from kinetochores and centromeres [64]. Thus, a ZW10-like function of CP103 in spindle assembly checkpoint regulation was refuted and the function of CP103 remains unknown. 

### 2.2. Composition of the Layered Core

#### 2.2.1. Outer Core Layers

The first core protein to be characterized in *Dictyostelium* was the NIMA-related kinase Nek2 [57]. It was identified by its high similarity to mammalian Nek2 in a cDNA project [206]. As in mammalian cells *Dictyostelium* Nek2 resides at the centrosome throughout the entire cell cycle [58]. At first glance this may sound surprising since in animal cells Nek2 kinase activity appears to be required only in late G2, to allow centrosome separation and the formation of two spindle poles (see above). However, independent of its kinase activity Nek2 appears to have also a structural purpose in centrosome biogenesis, possibly explaining its permanent centrosomal residency. In *Dictyostelium*, overexpression of both active and kinase-dead Nek2 caused centrosome amplification [57], and experiments with *Xenopus* extracts suggested a role in centrosome assembly also in animals [207]. The designation of *Dictyostelium* Nek2 as an outer core layer component is based on deconvolved confocal images, and its presence at mitotic centrosomes. *Dictyostelium* Nek2 kinase activity has so far been proven only using artificial substrates [208]. The hypothesis that the corona protein CP248 could be its primary substrate (see Section 2.1.3), would also be in agreement with its localization at the outer core layers. 

Two further outer core layer proteins, CP55 and Cep192, were identified through centrosomal proteome analysis. CP55 is the only core protein for which a complete knockout has been achieved [56]. CP55null cells exhibited impairment of centrosome splitting during prophase and often produced supernumerary MTOCs during telophase. Both effects could be related to the observed increase in ploidy. Furthermore, CP148 was recruited prematurely, i.e., already in metaphase instead of telophase. Whether this effect is causative for the formation of supernumerary MTOCs is unknown. These surplus MTOCs were clearly not centrosomes, neither regarding their ultrastructure nor their composition which included only corona components but lacked core proteins. Interestingly, CP55null cells grew in liquid culture, albeit slowly, but were unable to grow with bacteria as a food source. This phagocytosis defect could be based on their partially disorganized Golgi apparatus. Yet, the relationship between CP55 and the Golgi complex remains unknown.

Cep192 was identified in the centrosomal proteome and when expressed as a GFP fusion protein it was found at the core structure, and at spindle poles during mitosis [52,64]. Only recently we analyzed Cep192 localization and function more closely [54]. Using expansion microscopy it could clearly be assigned to the outer core layers. This superresolution technique also revealed a tight relationship with CDK5RAP2, which was confirmed by the mutual interaction of both proteins in BioID assays. BioID also revealed Cep192 interactions with all other known proteins of the layered core structure (see below). When overexpressed, GFP-Cep192 elicited supernumerary centrosomes, and Cep192 depletion destabilized the corona causing the appearance of supernumerary cytosolic MTOCs, similar to the CP55null phenotype. Taken together these phenotypes suggest that Cep192 is a key protein for the recruitment of corona components during centrosome biogenesis and is required for the maintenance of a stable corona structure. 

#### 2.2.2. Central Core Layer

Three proteins of the core structure, CP39, CP91 and CP75, were attributed to the central layer since they all disappear from mitotic centrosomes [33,53]. This attribution was later confirmed by ExM [54]. All three appear to be essential, since their depletion caused severe phenotypes, and knockout attempts failed altogether. 

Depletion of CP91 elicited supernumerary centrosomes and, as a consequence, defective chromosome segregation resulting in a heavily increased ploidy. Supernumerary centrosomes do not necessarily have this effect in *Dictyostelium*. As long as they remain cytosolic, they cannot interfere with bipolar spindle formation. The continuous presence of the nuclear envelope in semi-closed mitosis prevents the interference of microtubules nucleated by supernumerary centrosomes with kinetochores. Yet, nucleus-associated supernumerary centrosomes as observed in CP91-RNAi cells result in multipolar spindles and consequently, in aneuploidy [33]. Interestingly, overexpression of CP91 also elicited supernumerary centrosomes, however these were usually cytosolic and the effect on ploidy was much less severe. Taken together these results indicate that CP91 is required for centrosomal integrity and proper centrosome biogenesis. A further, independent function was also revealed by depletion of CP91, since the abscission process during cytokinesis was inhibited in CP91-RNAi cells. A role for centrosomal proteins in cytokinetic abscission is not without precedent. It has been shown very clearly for Cep55 and centriolin in animal cells [41,44]. 

BioID disclosed a mutual interaction between all three known central layer components, i.e., CP91, CP75 and CP39 [53]. CP39 is capable of homo-oligomerization and when overexpressed, it appears to act as a landing platform for further core components including CP75, CP91, Cep192 and CP55, which together form cytosolic pre-centrosomal clusters. In many cases, overexpression of CP39 culminated in the formation of supernumerary centrosomes capable of microtubule nucleation [53]. The conclusion was that in a similar manner, CP39 could recruit its partners during centrosome biogenesis, at the stage when the whole core structure is growing prior to splitting and loss of the central layer. Such an essential role also agrees with the observation that depletion of CP39 stopped growth completely. In a sense the properties and behavior of CP39 are reminiscent of Spc42p in budding yeast. Here Spc42p oligomerizes to form a crystalline array in the satellite (i.e., the precursor of the central plaque of the new spindle pole body) around which the whole new yeast centrosome is built [209,210]. 

CP75, the other binding partner of CP39 and CP91 is also involved in centrosome biogenesis. Here, supernumerary MTOCs were observed upon CP75 depletion [53]. Live cell imaging of GFP-tubulin in CP75RNAi cells suggested that they were caused by difficulties in spindle formation after centrosome splitting. Mitotic centrosomes frequently failed to organize an intranuclear central spindle. Instead, spindle behavior and the lack of intranuclear GFP-tubulin indicated that upon CP75 depletion, permeabilization of the nuclear envelope does not take place, and that mitotic centrosomes have difficulties to enter their fenestra within the nuclear envelope. Consequently, an extranuclear spindle was formed and chromosome segregation did not occur. This was in agreement with the strong increase in ploidy in CP75RNAi cells. Although depletion of CP75 caused the co-depletion of both CP39 and CP91 [53], the lack of mitotic nuclear envelope permeabilization was specific to CP75 and not observed upon depletion of either CP39 or CP91. The CP75RNAi phenotype raised the intriguing hypothesis that fenestration of the nuclear envelope upon centrosome duplication is the key event in permeabilization of the nuclear envelope during semi-closed mitosis. Forms of mitosis between fully open mitosis, as in most animal cells, and fully closed mitosis as in yeasts, cover a wide range from relatively open forms, in which the nuclear envelope no longer contains functional NPCs and is perforated by huge fenestrae, as for example in the *Drosophila* embryo [211], to fully intact mitotic nuclear envelopes in which only the dissociation of certain NPC components is sufficient to relieve the permeability barrier for large proteins, as for example in the fungus *Aspergillus nidulans* [212]. In *Dictyostelium* there are no indications of any fenestration of the nuclear envelope, in addition to the integration site of mitotic spindle poles. Our still unpublished results indicate that nuclear envelope permeabilization also occurs via partial disassembly of nuclear pore complexes, as in *Aspergillus* (I. Meyer and K. Mitic, unpublished). Yet, the frequent failure to organize an intranuclear spindle in CP75RNAi cells indicates that nuclear envelope fenestration during centrosome integration is the overarching event, which has to occur first. Exactly how fenestration happens in *Dictyostelium* is still unknown. It is tempting to assume a similar mechanism as in fission yeast *Schizosaccharomyces pombe*, which in spite of its closed mitosis is similar to *Dictyostelium* in that its spindle pole body remains cytosolic during interphase and enters the nuclear envelope only during mitosis [213]. Here the nuclear envelope membrane protein Brr6 drives insertion of the SPB into the nuclear envelope during mitosis [214]. Homologous proteins have been found in all organisms capable of forming nuclear envelope fenestrae for mitotic centrosomes, including *Dictyostelium*. Here, preliminary experiments indicated a presence of the Brr6 homologue at the nuclear envelope (M. Grafe unpublished results), but its function has not been elucidated. Future experiments will show whether and how the centrosome engages Brr6 and other membrane modifying components to achieve formation of the centrosomal fenestrae of the nuclear envelope. Of all known centrosomal components CP75 is certainly the most likely candidate for a key-role in this process. This also fits the observation that of all central layer components, CP75 is the last one to dissociate from mitotic centrosomes. It is still present after centrosome splitting [53], which occurs after fenestration [31]. 

## 3. Regulation of Centrosome Duplication and Mitotic Spindle Organization

We hypothesize that centrosome duplication proceeds as follows: Cep192 is major component of the outer layers, and the main centrosomal protein remaining after disintegration of the corona and dissociation of the central layer proteins. Cep192 then instantly recruits CDK5RAP2, possibly aided by CP55, which however plays a subordinate role since it can be knocked out completely. CDK5RAP2 then recruits γ-TuCs to organize the spindle. In late mitosis, upon progression of the folding process, Cep192 recruits CP39, which acts as a landing platform for CP75 and CP91. Afterwards CDK5RAP2 recruits CP148 and further γ-TuCs to build the new corona. This working model is based on our current knowledge of centrosomal substructures, their re-organization during mitosis and the characterized proteins. Of course, we still need more experimental proof to verify this model and to elucidate the regulation of these events.

### 3.1. Regulatory Kinases

Centrosome splitting and the concomitant dissociation of corona and central layer components is certainly a regulated process, and of course, mitotic kinases are the most likely (but not the only possible) regulators. In animal cells, the separation process of the two outer layers, and thus the splitting into two centrosomal entities, is reminiscent of the Nek2-dependent separation of the two centrosomal entities at the G2/M transition. Nek2 is a likely candidate regulator in *Dictyostelium* as well, by triggering the dissociation of phosphorylated targets both in the corona and the layered core. However, although Nek2 can be functionally expressed in and purified from both *E. coli* and *Dictyostelium* [57,208], to date no detailed investigation of the natural substrates of Nek2 has been performed. The three central layer proteins, CP39, CP75, CP91, and the corona component CP248, the putative orthologue of the human Nek2 target C-Nap1 (see above), are all candidates for Nek2 substrates, since all four proteins contain Nek2 target consensus sequences (predicted by ELM [215]) and leave the centrosome upon the splitting process.

Further Nek2 interactors could be phosphatases. In mammalian cells, Nek2 function is interconnected with protein phosphatase 2A (PP2A). PP2A is inhibited by CIP2A (inhibitor of PP2A), which in turn is an interactor of Nek2 [216]. Interestingly, another protein linked to PP2A function, phr2AB was found at the *Dictyostelium* centrosome and characterized as an interactor of CDK5RAP2 [138]. But based on the connection to PP2A, phr2AB could also be indirectly associated with Nek2. A further regulator of Nek2 is protein phosphatase 1γ (PP1γ), which counteracts Nek2 activity with its centrosomal substrates [217]. This regulatory complex is stabilized by the STE20-like kinase Mst2, which forms a ternary Nek2A-PP1γ-Mst2 complex. This complex is regulated at the G2/M transition by polo-like kinase 1 (Plk1), which phosphorylates Mst2 and destabilizes the complex. In the absence of PP1γ, Nek2 can effectively phosphorylate its centrosomal substrates and drive centrosome disjunction [218]. Mst2 and the closely related Mst1 are homologues of *Drosophila* Hippo, the name-giving kinase of the hippo pathway, which is essential for the regulation of organ growth and development [219]. In the on-status PDK1 (phosphoinositide-dependent kinase) forms a complex with Mst1/2, the scaffolding protein Sav (salvador) and LATS1/2 (large tumor suppressor kinase, homologous to *Drosophila* Warts). In this complex, LATS1/2 is activated by Mst1/2 and phosphorylates the transcriptional co-activator YAP (Yes-associated protein), which prevents cell growth. In the presence of growth factors PDK1 is recruited to the plasma membrane and the Hippo-complex dissociates, which turns off Hippo signaling [220]. Yet, Mst2 regulation of centrosome disjunction via Nek2 is independent of this canonical pathway, since it only involves Sav and Mst2, but not the other components including LATS1/2 or YAP [221]. 

With Nek2, PP1, SvkA (Mst1/2) and Plk, *Dictyostelium* expresses orthologues of the whole module regulating centrosome disjunction in mammals. SvkA was originally identified as a regulator of the F-actin severing protein severin, but the latter is not the primary target of SvkA. Interestingly, SvkA interacts with CDK2RAP2 [180], which was later shown to be true also in mammalian cells [222]. In *Dictyostelium* CDK5RAP2 negatively regulates SvkA and therefore also LATS, which was also found at the centrosome [152,180]. When fragments of CDK5RAP2 were overexpressed, the authors observed slow growth and delayed development. Since LATS1/2 are centrosomal proteins in mammalian cells as well [223], this pathway could be conserved and CDK5RAP2 could serve as a hub for its components at the centrosome. In neurons, loss of CDK5RAP2 reduced Hippo-dependent YAP/TAZ signaling, possibly affecting cell proliferation which would explain CDK5RAP2-dependent microcephaly [222]. 

Although SvkA, Nek2 and Plk have all been localized microscopically to the *Dictyostelium* centrosome and PP1 was identified in its centrosomal proteome [52], it is unclear whether there exists a Nek2, PP1, SvkA, Plk module to regulate centrosome splitting in a similar fashion as in mammalian cells (see above). The fact that knockout of the hippo orthologue SvkA interferes only with the abscission process during cytokinesis but not with centrosome duplication, argues against it being an essential component of the hypothetical module [160]. Yet, knockout of *Dictyostelium NdrC* (LATS), which is not part of the Nek2/PP1/Mst2/Plk1 module in mammalian cells, results not only in cytokinesis defects but also in centrosome amplification, supporting a role of hippo components in *Dictyostelium* centrosome biogenesis [152]. 

Two further, related STE20-like kinases, NdrA and SepA, were found also at the *Dictyostelium* centrosome [147,154]. Both proteins co-purified with isolated centrosomes. NdrA was absent from mitotic centrosomes, and this was independent of the phosphorylation state of its upstream regulator MST3. Surprisingly, knockout of NdrA had no obvious effects on centrosome integrity or its duplication, but rather it impaired phagocytosis. Since NdrA interacts with the Golgi-associated membrane protein EmpC and thus, is associated with vesicle trafficking, the authors concluded that a centrosomal signal originating from NdrA may regulate phagocytosis [147]. In addition to the phagocytosis defect of CP55null cells mentioned above (2.2.1.) [56], this is another indication that centrosomal proteins are involved in Golgi function and phagocytosis in *Dictyostelium.*

SepA was identified in a screen for cytokinesis mutants [154] and turned out as an orthologue of the Cdc7 kinase of the septation initiation network (SIN) that drives mitotic exit in *S. pombe* [224]. SepA’s upstream regulator, the small GTPase Spg1, localized to the centrosome as well. Based on the conservation of the SIN pathway proteins and the defects in cleavage furrow formation in SepA knockout cells, it became clear that these proteins are part of a conserved mitotic exit pathway but are not involved in centrosome duplication or required for centrosome integrity.

By contrast, in analogy to animal cells, Polo-like kinases (Plks), Aurora kinases, and cyclin-dependent kinases (CDKs) in addition to Nek2 are good candidates for regulators of the centrosome splitting process, including corona disassembly and dissolution of the central core layer.

Among the seven CDKs found in *Dictyostelium discoideum* [225] CDK1 is the best candidate, as it is active at the time of centrosome splitting. Polo-like kinases and Aurora kinases are represented in the *Dictyostelium* genome by only one member each, Plk and AurK, respectively. No centrosomal substrates are known for any of the abovementioned *Dictyostelium* kinases, however at least Plk and AurK have been localized at mitotic centrosomes and centromeres [64,115]. Despite its presence at mitotic spindle poles, a role of AurK in the centrosome splitting process appears less likely than for Plk, CDK1 and Nek2, since in contrast to these, Aurora kinases are not directly involved either in centriole duplication in animal cells, nor in spindle pole body duplication in yeast. The presence of various phosphorylation consensus sequences for Plk, CDK1 and Nek1 in CP39, CP75, CP91 and CP248, suggests that these dissociating centrosome components are promising substrate candidates for these kinases in the course of the splitting process. However, it should be kept in mind that phosphorylation of binding partners at the outer core layers could also be involved in the splitting process. Eventually we need to wait until the centrosomal kinase targets for the individual kinases will be identified.

### 3.2. Mitotic Spindle Organization and Spindle Elongation

In normal cells, centrosome splitting is inevitably coupled to spindle formation. Generally, spindle microtubules can be divided into two categories. Kinetochore microtubules connect spindle poles to kinetochores. Pole-to-pole microtubules which emanate from both poles and interdigitate in the equatorial region, connect both opposing poles [226]. Of course, the mitotic centrosomes are major nucleators and organizers of these microtubules, but in many species there are two additional pathways for microtubule nucleation. First, spindle microtubules can also be nucleated at chromatin. Here the associated guanosine nucleotide exchange factor RCC1 ensures a high concentration of the small GTPase Ran in its GTP-bound form. Ran-GTP then activates the microtubule-associated protein and spindle assembly factor TPX2, which in turn recruits Aurora A kinase to the spindle, where it activates further spindle assembly factors together with TPX2 [227,228,229]. The activated spindle assembly factors recruit γTuRCs, which in turn nucleate microtubules. The γTuRCs are only loosely bound to the chromatin, and with the aid of plus-end-directed chromokinesins the microtubules are arranged in a way that the minus-ends together with the γTuRCs become oriented towards the periphery, where they interact via dynein/dynactin with pre-existing microtubules emanating from the centrosome. Through the minus-end directed motor activity of dynein the γTuRCs can finally join the mitotic centrosomes [226]. This process augments the number of microtubules and greatly speeds up bipolar spindle formation. In many organisms there is a further centrosome-independent pathway to augment microtubule number during prometaphase, which is called the augmin-dependent pathway [38]. Augmin, also called HAUS complex, is an eight-subunit complex capable of binding laterally to pre-existing spindle microtubules, and of recruiting γTuRCs via NEDD1/GDP-WD (see above). TPX2 also plays a role in this process, since it binds to pre-existing microtubules before recruiting augmin [230,231]. Augmin activity strongly increases microtubule number and, thus, the probability that kinetochores capture microtubule plus ends. In parallel, γTuRCs at the minus ends are transported towards the poles via dynein and/or poleward microtubule flux. In centrosome-free cells such as in plants, or in many animals during oogenesis, the TPX2 and augmin-dependent pathways are the major spindle assembly pathways, and even in cells that do contain centrosomes, TPX2 and augmin-dependent spindle assembly strongly increases the efficacy of the whole spindle formation process [232].

Interestingly, orthologues of both TPX2 and augmin are absent in the *Dictyostelium* genome. We hypothesize that the absence of these components has co-evolved with the phenomenon of centromere clustering. In interphase *Dictyostelium* amoebae, all six subtelocentric centromeres are clustered in the pericentrosomal area of the nuclear matrix [233]. This cluster appears to be linked to the centrosome at the cytosolic side of the nucleus via a connector including the nuclear envelope protein Sun1 (see below). Centromere clustering ensures a close proximity of kinetochores to the centrosome at the onset of the spindle formation process. Thus, there is a high probability that the plus ends of new microtubules nucleated at the duplicating mitotic centrosomes will find their target kinetochores abolishing the need for acentrosomal TPX2 and augmin-dependent spindle assembly processes. The same holds true also for fission yeast, where the absence of genes encoding augmin and TPX2 is likewise accompanied by centromere clustering [234].

Once bipolar spindle formation is completed in metaphase, the cells transit into a continuous anaphase, in which the chromosomes migrate towards the poles, while at the same time the poles are separating from each other. The spindle assembly checkpoint, whose inactivation normally initiates anaphase, has been poorly investigated in *Dictyostelium*. The classic players, Mad1, Mad2, Cdc20, Bub1 and Bub3 are well conserved in the genome and, when labeled with GFP, Mad1 nicely accumulates at kinetochores during mitosis [235]. However, compared to many other cell types, the spindle assembly checkpoint seems to be prone to a phenomenon called “checkpoint slippage” [236], i.e., it can be overcome even when not all kinetochores are attached to microtubule plus-ends. This becomes evident when *Dictyostelium* amoebae are treated with microtubule-depolymerizing drugs such as nocodazole or thiabendazole. Although these drugs effectively inhibit spindle formation in prometaphase, they suppress cell cycle progression only for a few hours before cells re-enter interphase containing a single nucleus with increased ploidy. This is usually fatal upon entry into the next cell cycle, as these drugs do not affect centrosome duplication and, thus, the cells enter interphase with an additional, nucleus-associated centrosome, which also duplicates at the next G2/M transition. The resulting formation of multipolar spindles usually yields inequal chromosome segregation and finally cell death (see Section 2.2.2).

Anaphase and telophase are characterized by a continuously increasing distance between the two spindle poles. As these movements are accompanied by elongation of the central spindle, it was originally assumed that as in many other cells, spindle elongation is the driving force for spindle pole separation. However, this simple view was refuted after Koonce and co-workers observed spindle pole separation in a mutant unable to form a central spindle [237]. These cells carry a knockout of the gene encoding Ase1, a member of the ubiquitous MAP65/Ase1/PRC1 family of MT crosslinkers [238]. These are microtubule-bundling proteins serving crosslinking and spindle elongation in the spindle midzone, where pole-to-pole microtubules interdigitate. Consequently, Ase1 which is sequestered in nucleoli during interphase, strongly accumulates at the spindle midzone during mitosis. Ase1 knockout cells are viable and will complete mitosis, obviously because they are able to separate the two daughter nuclei solely by pulling forces exerted via astral microtubules, most like through minus-end directed motor activity of cortical dynein [237].

## 4. Centrosome-Nucleus Attachment

**Figure 4:**
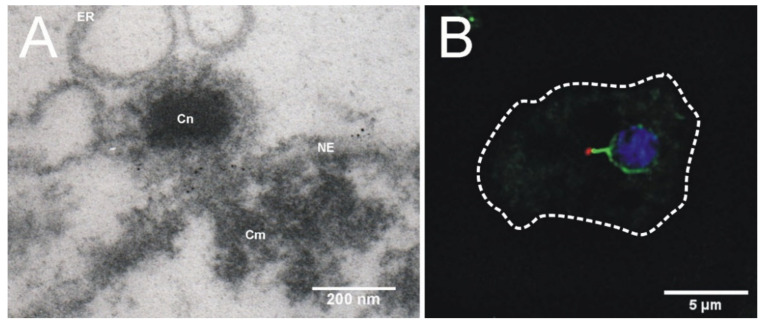
Centrosome-Nucleus-Centromere cluster. (**A**) Immunoelectron microscopy image showing one section of an isolated nucleus with the attached centrosome. Nuclei were labeled with an antibody against *Dictyostelium* Sun1 and nanogold conjugated anti-rabbit antibodies. The centrosome (Cn), the centromeric cluster (Cm), the nuclear envelope (NE) and the endoplasmic reticulum (ER) are indicated (image by Prof. Otto Baumann); (**B**) Immunofluorescence microscopy image of a Sun1-GFP knock-in cell (green) stained with an antibody against the centrosomal core protein CP91 and anti-rabbit-AlexaFluor 568 conjugates (red) and DAPI (blue). The cell edges are outlined by a dashed line. Taken from [243].

Like all centrosomal structures in vegetative cells, the *Dictyostelium* centrosome is structurally linked to the cytosolic side of the nucleus during interphase. Not surprisingly, one key protein of this linkage is the nuclear envelope protein Sun1, named after the founding members of the Sun-family, i.e., fission yeast Sad1 and *Caenorhabditis elegans* UNC-84, which share a common Sun-domain. In most eukaryotes Sun1 is an inner nuclear membrane protein, forming a trimer and interacting, via its Sun-domain, with the so-called KASH-domain proteins (named after Klarsicht, ANC-1, SYNE1 homology) within the perinuclear space [239]. Since the various KASH domain proteins interact directly or indirectly with all three cytoskeletal elements (actin, microtubules, intermediate filaments) the term LINC complex (linker of the nucleus and cytoskeleton) was coined for the Sun/KASH domain protein heterodimer [240]. At the nuclear side, Sun1 interacts with lamins in animal cells and also in *Dictyostelium* [241]. Yet, on the cytosolic face of the nuclear envelope the situation in *Dictyostelium* seems to be unique. Sun1 is present in both nuclear membanes with no strong bias towards the inner nuclear membrane [124,125] and there is no clear orthologue for a KASH domain protein. Due to its similarity to mammalian nesprins, the outer nuclear membrane protein interaptin was discussed as a *Dictyostelium* KASH domain protein [125,242]. But interaptin is certainly no part of a LINC complex, as it lacks the conserved KASH domain and obviously does not interact with Sun1 [125]. Sun1 is however required for centrosome/nucleus attachment. It co-purifies with isolated centrosomes and is concentrated at the nuclear envelope in the direct vicinity of the centrosome (Figure 4). Sun1 mutants are defective in centrosome/nucleus attachment. It is possible that the centrosome/nucleus linker employs Sun1 on both sides of the membrane, and that an unknown protein of the perinuclear space mediates this interaction. Although a direct interaction with Sun1 remains to be proven, the unusual kinesin Kif9 is a likely candidate for a LINC complex component in *Dictyostelium*. Kif9 is an internal motor kinesin, which can be grouped into the kinesin-13 family, which usually act as microtubule depolymerases [130]. Within this group Kif9 is unique in containing a ~23 residue transmembrane domain close to its C-terminal end, targeting the protein to the outer nuclear envelope where it accumulates in the pericentrosomal region. Knockout of Kif9 disrupts the centrosome/nucleus linkage and causes dispersal of Sun1, away from the pericentrosomal region of the nuclear envelope [130].

Thus, Sun1 and Kif9 are likely to form a complex. It is possible that microtubule binding by the Kif9 motor domain coupled to its microtubule depolymerizing activity exerts a pulling force on the centrosome, bringing it closer to the nucleus. A direct interaction between Sun1 and a kinesin would be without precedent, but an indirect interaction of Sun1 with kinesin-1 via a KASH-domain protein is well established in several species [244].

Kinesins are not the only motor proteins involved in centrosome/nucleus attachment. Dynein too is linked to KASH domain proteins in yeasts, animals and most likely also in *Dictyostelium* [244]. This is based on the observation that a hypomorphic mutation in the dynein regulator Lis1 causes centrosome detachment from the nucleus [103]. Dynein may function together with Kif9 to bring the centrosome close to the nucleus through its microtubule minus-end directed motor activity. Whether and how Lis1 and dynein interact with Sun1 in this context is not known.

Despite the tight relationship between the *Dictyostelium* centrosome and Sun1, the Sun1 binding partners at the centrosome are still unknown. Currently there are three candidates based on observed mutant phenotypes, i.e., the corona proteins CP248, CP148 and CenB. CP248 must be somehow related to Sun1 since localizations of Sun1 and, interestingly, also interaptin at the nuclear envelope are both reduced in CP248 knockout cells [57]. A role of CP148 in centrosome/nucleus attachment was proposed based on the observation that in CP148 RNAi cells, centrosomes were frequently found detached from the nucleus [50]. A similar phenotype was also observed upon knockout of centrin B [116]. Yet, in all these cases it remains elusive how these proteins are employed in centrosome/nucleus attachment. The fact that the centrosome remains nucleus associated even after loss of the corona in prophase, may also indicate a role of core layer proteins in centrosome/nucleus attachment.

## 5. Conclusions

Research into the *Dictyostelium* centrosome during the last twenty-five years has revealed a fairly detailed picture of its structure, organization and dynamics. As expected for this ancient organelle, many similarities with the various centrosome types of animals and fungi emerged, especially regarding the organization of microtubule nucleation complexes and the proteins involved. However, as reflected also by structural differences, most prominently the lack of centrioles, there are clear differences in centrosome duplication and its regulation. Comparative studies of centriole-containing vs. acentriolar *Dictyostelium* centrosomes nicely revealed several basic, centriole-independent functions, including not only microtubule organization, but also cytokinesis and Golgi function. Future directions will focus on the elucidation of the centrosome’s role in nuclear envelope dynamics during semi-closed mitosis, and on the still not well understood regulation of the dynamic processes during its duplication.

## Figures and Tables

**Table 1:** Proteins localized at *Dictyostelium* centrosomes.

Amoebozoa *Dictyostelium*	Opisthokonta Metazoa *Homo sapiens*	Opisthokonta Fungi *S. cerevisiae*	Opisthokonta Fungi *S. pombe*	Archaeplastida *Arabidopsis thaliana*	Excavata *Trypanosoma spec*.	SAR *Plasmodium falciparum*, *Albugo spec*.
**Central layer(s)**						
CP91 [33]	-	-	-	-	-	-
CP75 [53]	-	-	-	-	-	-
CP39 [53]	-	-	-	-	-	-
**Outer core layer**						
Cep192 [54]	Cep192/SPD2 [55]	-	-	-	-	-
CP55 [56]	-	-	-	-	-	-
Nek2 [57]	Nek2 [58]	Kin3p [59]	Fin1 [60]	AtNek2 [61]	TbNRKC [62]	Pfnek-2 [63]
CP44 [64]	-	-	-	-	-	-
**Corona**						
γ-tubulin [65]	γ-tubulin [5]	Tub4 [66]	gtb1 [67]	γ-tubulin [68]	γ-tubulin [69]	γ-tubulin [70]
Spc97 [65]	GCP2	Spc97 [66]	Alp4 [67]	GCP2 [68]	GCP2 [69]	GI: 389585322
Spc98 [65]	GCP3	Spc98 [66]	Alp6 [67]	GCP3 [68]	GCP3 [69]	GI: 389585419
CDK5RAP2/Cep161 [71]	CDK5RAP2/Cep215/Cnn [72]	Spc72p [73]	Mto1/Mbo1/Mod20 [74]	-	GI: 407424972	GI: 23479271
CP148 [75]	Pericentrin/PCNT/PLP [76]	Spc110p [73]	Pcp1 [77]	-	-	GI: 325186828
TACC [78]	TACC/Maskin [79]	-	Alp7/Mia1	GI: 297312240	- *	GI: 325183149
CP224 [80]	chTOG/XMAP215 [81]	Stu2p [82]	Dis1/Alp14 [83]	MOR1 [84]	XMAP215 [85]	GI: 1976646509
EB1 [86]	EB1 [87]	YEB1/Bim1p [88]	Mal3 [89]	EB1c [90]	EB1	EB1
Moe1 [91]	eIF-3 subunit 7	-	Moe1 [92]	eIF-3 subunit 7	eIF-3 subunit 7	eIF-3D
CP248/CP250 [64,93]	C-Nap1/Cep250 [94]	-	-	-	-	-
CenA/DdCrp [95]	Centrin-3 [96]	Cdc31p [97]	Cdc31 [98]	Centrin [99]	Centrin [100]	Centrin [101]
CP103 [64]	-	-	-	-	-	-
**Corona-associated**						
Dynein DHC [102,103]	DHC [104]	Dyn1p [105]	Dhc1 [106]	-	DHC [107]	DHC [108]
Dynactin (including p50, p62, Arp1/Centractin) (own unpubl [109])	Dynactin [110]	Dynactin [111]	Dynactin [106]	-	Dynactin	Dynactin [112]
Lis1 [103]	Lis1/PAFAH1B1 [113]	Pac1p [114]	-	-	SMU1	GI: 1678234918
**Centrosome-associated (no sublocation determined)**						
AurK [115]	AuroraA/B/C [116]	Ipl1p [117]	Ark1 [118]	ATAUR1/2/3	TcAUK1/2/3 [119]	Pfark-1/2/3 [120]
Plk [64]	Plk1 [121]	Cdc5p [121]	Plo1 [122]	-	TbPLK [123]	- [123]
Sun1 [124,125]	Sun1/Matefin [126]	Mps3p [127]	Sad1 [128]	Sun1 [129]	GI: 686631607	GI: 221061315
Kif9 [130]	Kif9 [131]	-	-	-	-	-
Kif12 [132]	Kif12 [133]	-	-	Kinesin-12	-	-
Nup53 (Meyer in prep)	Nup35 [134]	Nup53p [135]	Nup40 [136]	NUP35 [137]	TcCL_ESM01172	GI: 325183342
phr2AB [138]	PPP2R5D [139]	Cdc55p [140]	Pab1 [141]	AtB beta [142]	NCBI: XP_829543	phr2AB
HSBP1 [143]	HSBP1 [144]	-	-	AtHSBP [145]	-	HSBP [146]
NdrA [147]	NDR1 [148]	Cbk1p [149]	Orb6 [149]	AGC^§^ [150]	PK50/PK53 ^§^ [151]	AGC/AKT ^§^
NdrC [152]	LATS2	Dbf2p [149]	Sid2 [149]	AGC^§^ [150]	PK50/PK53 ^§^ [151]	AGC/AKT ^§^ [153]
SepA [154]	-	Cdc15p [155]	Cdc7 [156]	-	-	-
Spg1 [154]	-	Tem1p [157]	Sid3 [158]	AtSGP1 [159]	-	-
SvkA/Hrk-Svk [160]	MST1/2 [161]	Kic1p [162]	Sid1 [158]	SIK1 [163]	GI: 1919796340	- ^$^ [164]
NumA1 [165]	BRCT domain proteins ^&^	BRCT domain proteins ^&^	BRCT domain proteins ^&^	BRCT domain proteins ^&^	BRCT domain proteins ^&^	BRCT domain proteins ^&^
fttB [52]	14-3-3					
Clathrin light chain (clcA) [166]	CLC [167]	Clc1p [168]	Clc1 [169]	CLC2 [170]	TcClc [171]	GI: 124809181
AbpF [172]	-	-	-	-	-	-
AdcA [173,174]	b-arrestin 2 [175]					

Alternative names separated by “/”, not identified: “-”; * none with C-terminal TACC domain. Sequences with the TACC pfam domain exist, however without striking aa identity to TACC orthologues; ^§^ several paralogues, exact assignment difficult; ^$^ BLAST with SvkA as a query reveals one kinase annotated as STE/STE20 protein kinase (NCBI: ETW20826). However, Ward et al. concluded that there are no kinases of this family in the *Plasmodium falciparum* genome (which was complete at that time) [164]; ^&^ The BRCT (breast cancer carboxy-terminal) domain is found in several proteins and is conserved in all eukaryotic clades. BRCT-domain proteins share only a conserved 76 aa region, but it is not possible to judge whether *Dictyostelium* NumA1 has orthologues among the BRCT-domain proteins of other species.
